# Imaging of Cartilage and Chondral Defects: An Overview

**DOI:** 10.3390/life13020363

**Published:** 2023-01-28

**Authors:** Neha Nischal, Karthikeyan P. Iyengar, Deepak Herlekar, Rajesh Botchu

**Affiliations:** 1 Department of Musculoskeletal Radiology, Royal Orthopedic Hospital, Birmingham B31 2AP, UK; 2 Department of Radiology, Holy Family Hospital, New Delhi 110025, India; 3 Department of Orthopaedics, Southport and Omskirk Hospital, Southport PR8 6PN, UK; 4 Department of Orthopaedics, University Hospitals of Morecambe Bay NHS Foundation Trust, Kendal LA9 7RG, UK

**Keywords:** cartilage imaging, chondral defects, MRI, osteoarthritis

## Abstract

A healthy articular cartilage is paramount to joint function. Cartilage defects, whether acute or chronic, are a significant source of morbidity. This review summarizes various imaging modalities used for cartilage assessment. While radiographs are insensitive, they are still widely used to indirectly assess cartilage. Ultrasound has shown promise in the detection of cartilage defects, but its efficacy is limited in many joints due to inadequate visualization. CT arthrography has the potential to assess internal derangements of joints along with cartilage, especially in patients with contraindications to MRI. MRI remains the favored imaging modality to assess cartilage. The conventional imaging techniques are able to assess cartilage abnormalities when cartilage is already damaged. The newer imaging techniques are thus targeted at detecting biochemical and structural changes in cartilage before an actual visible irreversible loss. These include, but are not limited to, T2 and T2* mapping, dGEMRI, T1ρ imaging, gagCEST imaging, sodium MRI and integrated PET with MRI. A brief discussion of the advances in the surgical management of cartilage defects and post-operative imaging assessment is also included.

## 1. Introduction

The imaging of articular cartilage has raised immense interest and promoted research owing to the large prevalence of osteoarthritic (OA) joint disease, with cartilage degeneration being an integral part of the disease process. The pooled global prevalence of knee OA was found to be 16% by Cui et al. [1], with its incidence increasing with the ageing population. Our understanding of the microstructure, biomechanics as well as disease processes affecting articular cartilage has made leaps and bounds along with the advances in management techniques. The focus is now shifting to the initiation of therapy in early reversible stages of the disease. This has also translated into advances in imaging, which forms an integral part of OA management. Apart from detection, quantification and prognostication, imaging, especially MRI, is instrumental in the post-intervention assessment of treatment responses or the detection of complications. Radiologists must keep abreast of these advances for a better understanding and ease of communication with referring clinicians.

## 2. Anatomy

Articular cartilage is a specialized hyaline cartilage covering the joint surfaces and providing smooth gliding surfaces. It is devoid of vessels, nerves and lymphatics, which leads to a very limited reparative potential [2]. Cartilage turnover is also very slow, as chondrocytes divide slowly. 

Embryologically, chondrogenesis occurs from the condensed mesenchymal blastema which secretes the extracellular cartilage matrix and generates cells called chondroblasts. The mature cells caught in this abundant matrix are called chondrocytes [3,4]. The major components of articular cartilage and their functions are summarized in Table 1.

### Microscopic Structure

**Figure 1:**
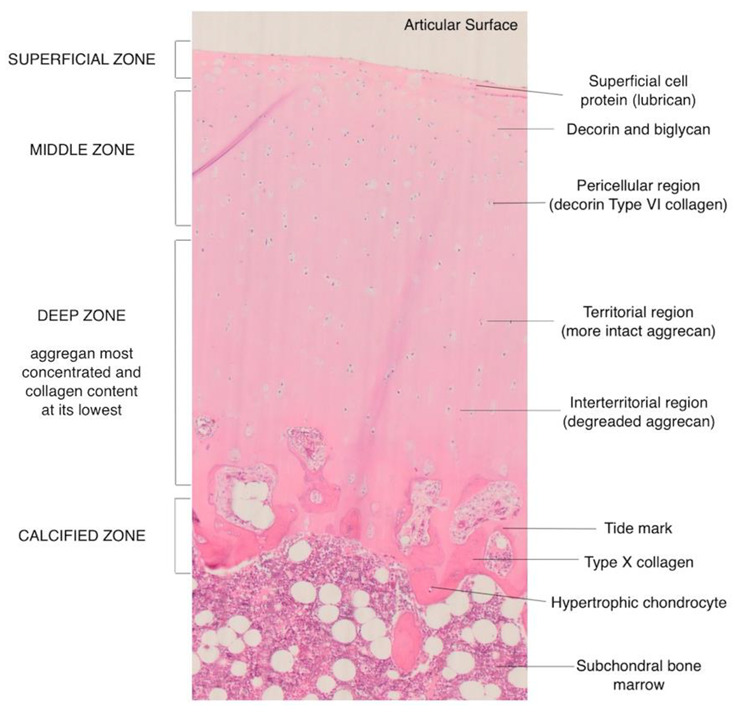
Histology image showing the different layers of the articular cartilage.

The cartilage is arranged into four layers or zones [2,3,5] (Figure 1).

1. Superficial or tangential zone. This is the thinnest layer which is adjacent and parallel to the joint surface and is made up of flattened cells. With the highest water content and greatest tensile strength, the integrity of this layer is crucial to prevent osteoarthritis.
2. Transitional or intermediate zone. Spheroidal cells are found in this zone along with randomly oriented collagen fibers.
3. Middle zone. The lowest cell density with the highest proteoglycan content is found in this layer. The cells are arranged in a perpendicular orientation to the surface.
4. Calcified cartilage zone. It is a mineralized zone acting as a shock absorber along with the subchondral bone. Owing to the low number of cells in a calcified matrix, there is a very low metabolic activity in this zone.

## 3. Cartilage Imaging

### 3.1. Radiographs

Conventional radiography is the most widely used imaging technique for the assessment of joint abnormalities. Although the articular cartilage is not directly visualized, it provides an indirect evaluation of cartilage integrity by measuring the joint space. There are limitations to this technique, as in cases of early cartilage loss, the radiographs show no loss of the joint space, and there maybe interobserver variations in assessing early OA. Another pitfall of using this method is the assumption that the joint space only consists of the articular cartilage, and thus joint space loss is secondary only to cartilage loss. While this may occur for hip joints and radiocarpal joint, it does not hold true for knee joints, where the joint space includes the menisci as well. The degree of knee flexion also affects the perceived joint space, leading to false positive or false negative interpretations. 

**Figure 2:**
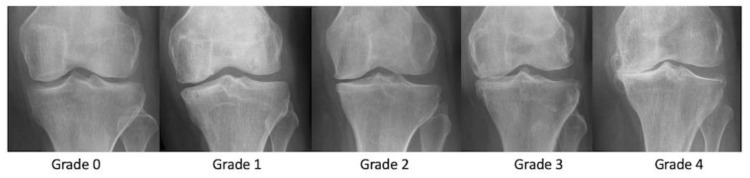
Anteroposterior radiograph of the left knee showing Kellgren–Lawrence grade 0, 1, 2, 3, and 4 OA.

Nevertheless, radiography is the least expensive, most widely available and reproducible imaging technique for this purpose. It serves as a baseline investigation as well as for the follow-up of disease progression or post-operative assessment. Osteochondral defects can be detected on radiographs as small fragments of subchondral bone. The Kellgren and Lawrence classification widely used for OA is based on radiographs; grades II–IV show a progressive loss of the joint space (Figure 2). Calcification of the hyaline cartilage can be seen in cases of chondrocalcinosis and is used as an adjunct diagnostic element, making the technique even better than MRI for this purpose.

*Diffraction-enhanced X-ray imaging* is an experimental novel technique that can provide the direct visualization of articular cartilage. It uses a series of silicon crystals to obtain highly collimated monochromatic X-rays which provide diffraction-enhanced images. Mollenhauer et al. showed these images of the articular cartilage to be comparable to gross and histological samples from intact as well as disarticulated joints [6].

### 3.2. Ultrasound 

Ultrasound is a robust modality for diagnosing various musculoskeletal pathologies. Its advantages include, but are not limited to, high resolution, wide availability, affordability, a dynamic nature and higher patient comfort. The efficacy of ultrasound for the evaluation of articular cartilage has been examined by various researchers over the past many decades. In 1984, Aisen et al. studied the femoral articular cartilage at the knee and found that in patients with arthritis, there was loss of sharpness of the articular cartilage on ultrasound, a finding which correlated with the clinical status and preceded the full-thickness loss of the cartilage [7]. Since then, there have been remarkable technological advances in ultrasound. Many other researchers have found ultrasound to be a valid tool for assessing articular cartilage when compared to histology [8,9]. 

**Figure 3:**
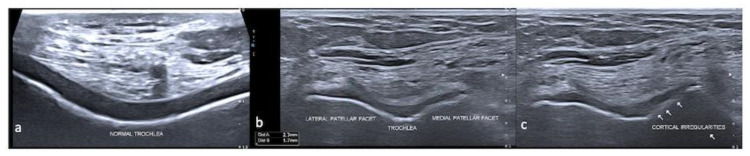
Ultrasound. Axial image showing the normal cartilage of the trochlea (**a**), thinning of the articular cartilage of the medial facet of the trochlea (**b**) and cortical irregularity (**c**).

The normal articular cartilage appears homogenously anechoic owing to its inherent high water content, with smooth, sharp margins (Figure 3). Owing to a clear interface with the overlying soft tissues and underlying bone, its thickness can be measured and varies from 0.1 to 0.5 mm in the hand to about 3 mm in the knee [10,11,12]. Its evaluation requires appropriate patient and probe positioning so as to obtain a good acoustic window to visualize the cartilage. However, in deep joints, it may not be possible to evaluate the entire cartilage, and the assessment is all the more difficult in pathological joints as the patient may not be able to flex or extend the joints due to pain. The presence of osteophytes can also obscure the cartilage due to acoustic shadowing. Another limitation of this modality is operator dependence and a steep learning curve.

### 3.3. CT/CT Arthrogram

Computed Tomography (CT) is not typically used for the evaluation of articular cartilage but can be used where MRI is contraindicated. 

**Figure 4:**
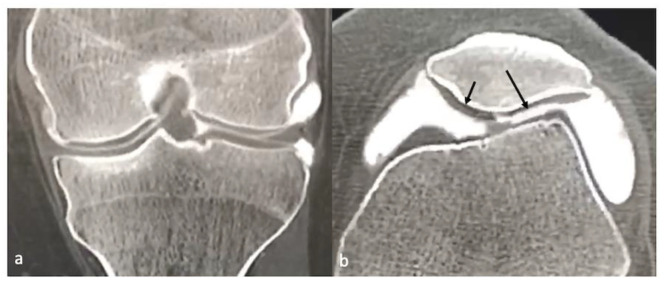
CT arthrogram of the knee coronal (**a**) and axial (**b**) planes showing normal cartilage (small arrow) and focus of partial thickness cartilage defect (long arrow).

CT arthrography (CTA) is performed by the intra-articular injection of contrast, and this can delineate the cruciate ligaments and menisci well, the downsides being the invasive nature of this technique and radiation exposure. CTA can also demonstrate normal cartilage as well as its defects as intra-articular iodine provides excellent contrast between the cartilage and surrounding high attenuating iodine based contrast [8,13] (Figure 4). Recent research has been targeted at evaluating the GAG content of cartilage using CTA. In this technique, after intra-articular injection of an iodine-based CT contrast, the patient is asked to move the joint actively. After some time, the scanning will show the contrast concentrated in the cartilage, as iodine is negatively charged. Studies have shown that the concentration of the contrast is higher in GAG-depleted cartilage; this method is known as quantitative CTA [14].

Although not widely in used at present, this technique offers certain advantages over MRI, including a shorter scan time, a lower cost and an alternative for patients with contraindications to MRI. 

**Figure 5:**
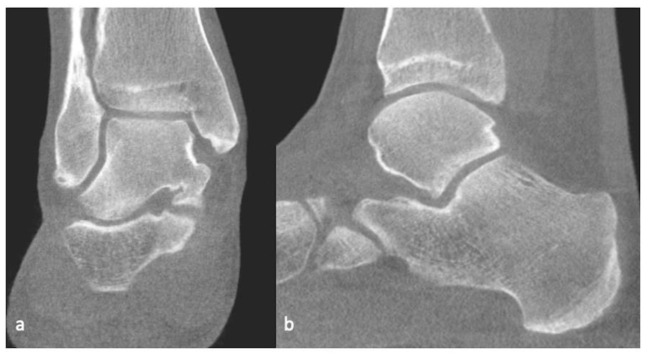
Weight-bearing CT, coronal (**a**) and sagittal (**b**) planes of the ankle.

Dedicated cone beam CT (CBCT) scans have been developed for musculoskeletal imaging [15,16]. These have been shown to be comparable or even superior to routine multidetector CT (MDCT) for bone and soft tissue evaluation, with the added advantage of the ability to perform weight-bearing scans, which is particularly useful for lower limb joint evaluation. Although direct cartilage visualization was found to be slightly better for MDCT, the ability of image acquisition during weight bearing using CBCT along with reduced radiation might favor this modality in future research [16]. Weight-bearing CT, which is predominantly used for foot and ankle, can also help to assess articular cartilage and joint alignment (Figure 5).

### 3.4. MRI 

Magnetic Resonance Imaging is the modality of choice for evaluating pathologies of articular cartilage. It shows an intrinsic high soft tissue contrast and can provide a non-invasive global assessment of the entire cartilage. Image quality is better at higher field strengths, and dedicated cartilage imaging is preferably performed with 3T or 1.5T scanners [17]. Various imaging sequences and advanced imaging are now available, in practice and in research settings, to assess joint morphology as well as cartilage composition. The rationale for this is to assess surgical or clinical treatment responses, as MRI can provide important clues regarding disease progression as well as the stability of a surgical repair. 

#### 3.4.1. Morphological Sequences

The basic MRI sequences used to characterize cartilage lesions in terms of size, location and depth include spin echo (SE) and gradient echo (GRE) images, with their modifications. 

**Figure 6:**
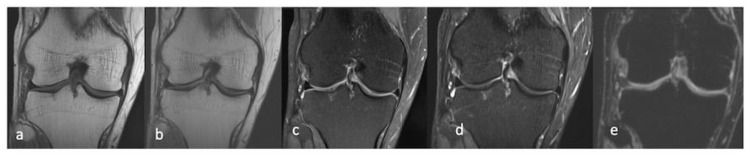
Coronal MRI- T1 (**a**), PD (**b**), PDFS (**c**), STIR (**d**), T2 med volume (**e**) of the right knee.

The standard SE or fast SE sequences include T1-, T2- and proton density- (PD) weighted images. PD with or without fat suppression is widely used in musculoskeletal imaging. In addition, 2D or 3D PD- as well as T2-W sequences provide good contrast of the articular cartilage from the adjacent structures, which is enhanced in cases of joint effusion (Figure 6).

Gradient-recalled echo (GRE) sequences render the cartilage hyperintense and are probably more useful in localizing loose bodies than the SE sequences. They may, however, overestimate cartilage thickness [18].

Moreover, 3D sequences without slice gaps enable isotropic image acquisition and are more sensitive in detecting small cartilage defects. These 3D sequences may be SE sequences such as SPACE or GRE sequences such as MEDIC. 

#### 3.4.2. Compositional Sequences

It is well known that biochemical changes occur much earlier than structural defects in the cartilage, and if these can be detected and an appropriate therapy initiated, it may be possible to halt or possibly reverse disease progression. Many innovative MRI techniques have been developed and are being researched to assess the GAG content and collagen framework. Some of these are discussed here.

##### T2 and T2* Mapping 

Both T2 and T2* can detect changes in hydration, collagen content and organization of the extracellular matrix. While T2 mapping is SE-based, T2* is GRE-based and is thus more susceptible to magnetic field inhomogeneities. However, T2* uses shorter echo times (TE) and thus enables reduced scan times with higher resolution [18,19,20,21]. 

The basic principle is the acquisition of T2 images at varying echo times (TE) using an exponential decay curve to calculate the decay time constant between signal intensity and TE. A healthy cartilage shows a laminar appearance with short T2 values in the superficial zone, higher values in the transitional zone with randomly oriented fibers and shorter values again in the deeper zone with tightly packed fibers in a perpendicular orientation. A degenerated cartilage, on the other hand, shows increased T2 values owing to the disruption of the fibrillar arrangement and changes in hydration. T2* mapping appears to be more sensitive in the detection of these changes than T2, with values that vary much later in the disease process. The variation in the relaxation times have, however, been found to be higher for T2 as compared to T2* in a study by Mars et al. [21]. The results are better for MRI at higher strengths (3T) as compared to MRI at lower strengths (1.5T). 

##### Gd-Enhanced MRI (dGEMRI) 

Delayed gadolinium- (Gd) enhanced MR imaging of cartilage (dGEMRIC) is based on the fact that GAGs, being negatively charged, inhibit the diffusion of gadolinium-based contrast agents (Gd-DTPA^2-^), which also carry a negative charge, into healthy cartilage. With degeneration, there is a progressive loss of the GAG content in cartilage, which facilitates the Gd uptake. The contrast, which is injected intravenously, diffuses into the synovial fluid and is then taken up by the degenerated cartilage. This leads to reduced T1 relaxation times, which are quantitatively assessed by T1 relaxometry, providing the so-called dGEMRIC index [18,19]. Color-coded spatial maps are used to depict the distribution of the T1 relaxation times. Since cartilage is avascular, and gadolinium uptake is based on diffusion, a scan delay of 90 min after intravenous contrast is recommended by many authors. 

Fleming et al. found a significant reduction in the dGEMRIC indices of the medial tibiofemoral compartment in ACL injured knees as compared to contralateral uninjured knees, suggesting the presence of degenerative changes in these patients [22]. 

The follow-up post-operative evaluation of the hip joints in patients who underwent surgery for femoroacetabular impingement revealed reduced dGEMRIC values in these patients, compared to the ones who did not undergo surgery [23].

This technique has also been investigated to evaluate the treatment response after autologous chondrocyte implantation. Watanabe et al. related the GAG concentration in biopsy specimens to the difference (ΔR1) between the relative pre- (R1_pre_) and post-contrast (R1_post_) relaxation times of repaired as well as normal cartilage. They found a significant correlation between ΔR1 and the relative GAG concentration (GAG concentration in repaired cartilage divided by that in normal cartilage) [24]. 

##### T1ρ Imaging

T1ρ is the spin lattice relaxation time in a rotating frame. It measures the motion of protons in their macromolecular environment and is said to be related to the PG content, with a loss of PGs leading to an increase in T1ρ. It has shown promise in the detection of early osteoarthritic changes in the knee and in assessing the menisci as well as hip joint cartilage, especially in cases of femoroacetabular impingement [13,19,25,26,27,28,29]. While the advantages of T1rho imaging include its sensitivity to early degeneration and ability to image without the administration of a contrast agent, the need for special sequences with limited availability and the long acquisition times are some practical limitations [13].

##### Sodium MRI

Sodium MRI imaging takes advantage of the attraction of positively charged Na^2+^ cations to the negatively charged GAGs; thus, Na^2+^ concentration is higher in the cartilage extracellular matrix than in the synovial fluid. The specific resonance frequency is measurable by MRI owing to the nuclear spin momentum of the sodium ions. While this technique has shown promise for detecting early OA changes and can be performed without administering a contrast agent, its clinical application is limited. This is because the concentration of sodium is lower than that of protons, leading to low a SNR and thus the need for special hardware, including a high field strength, optimized protocols and dedicated coils, which are limited in availability [13,18,19,30,31]. Other challenges in imaging include partial volume averaging because of the presence of synovial fluid, requiring fluid suppression techniques.

##### gagCEST

Glycosaminoglycan chemical exchange saturation transfer uses the presence of free and bound pools of protons to create an intrinsic contrast. The protons in the bound pool are associated with GAGs and can thus be used as biomarkers for their presence. This has shown good results when combined with other compositional techniques such as T1rho and sodium imaging [32,33]. There are, however, a number of challenges, such as an extreme sensitivity to magnetic field inhomogeneities, a long scan time, a need for a ultra-high field strength (typically 7T). Limited research and lack of data on the overall assessment of disease burden and treatment response preclude the widespread usage of this technique in the present scenario.

### 3.5. Integrated PET–MRI 

**Figure 7:**
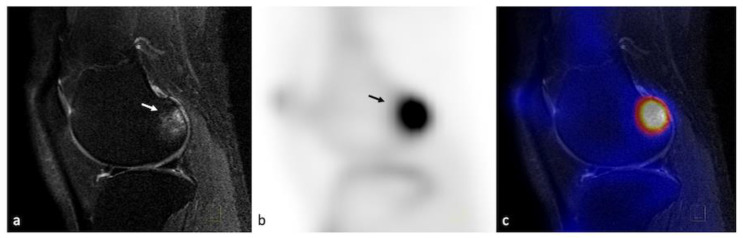
PET–MRI of the right knee of a 41-year-old female having Kellgren–Lawrence grade 2 OA, showing great signal intensity changes (arrow) in the lateral posterior femur on a T2 SPIR image (**a**) with a high tracer uptake on the sagittal PET image (arrow), (**b**) also spatially correlated on the fused PET–MRI image (**c**).

The principle of integrating Positron Emission Tomography (PET) with MRI is to understand the disease process at a molecular level before structural or compositional changes occur. The radioactive tracers used in the evaluation of joint pathologies, especially OA, include FDG (2-18F-fluoro-2-deoxy-D-glucose) and 18F-NaF (fluorine18-sodium fluoride). FDG, which denotes areas of increased glucose metabolism and thus cellular response, is shown to be increased in the joint space and around the knee joint, indicating cartilage damage and resulting inflammation. A high uptake is also seen in areas of bone marrow lesions detected on MRI [34]. 18F-NaF, on the other hand, is a marker of bone metabolism, showing a high and rapid uptake in areas of mineralizing bone. It has shown high uptake values in patients without radiographic evidence of joint space narrowing and correlates well with pain in patients with early or progressive OA [35]. Thus, fusing PET images with the unparalleled soft-tissue contrast of MRI to provide molecular-level data holds great promise to understand the complex disease process affecting articular cartilage [36] (Figure 7).

## 4. Pathologies 

**Figure 8:**
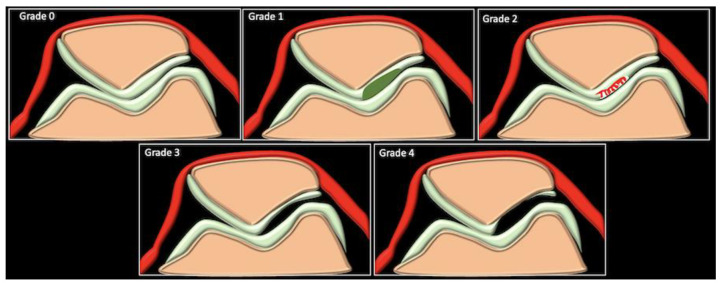
Schematic of the Outerbridge classification of chondral pathologies.

Cartilage injuries are broadly traumatic or degenerative in etiology. The modified Outerbridge classification is the most commonly used system for MRI-based grading of chondral degeneration or defects in a clinical scenario. It was originally developed based on the arthroscopic evaluation of the patellar cartilage but has now been extended to grade chondral lesions in all locations (Figure 8).

Grade 0 corresponds to the normal appearance of the articular cartilage, with normal signal intensity. Grade I lesions include focally increased signal intensity in the fluid-sensitive sequences without any visible cartilage loss or defect. Grade II lesions include fraying of the articular cartilage surface. Grade III lesions involve partial-thickness articular cartilage defects. Grade IV lesions involve full-thickness chondral defects with exposed subchondral bone and reactive bone changes. This correlates well with the arthroscopic scoring of chondral defects suggested by the International Cartilage Repair Society (ICRS). The grading helps in surgical planning and also has prognostic implications. Various authors have found patients with higher grade (III and IV) chondral injuries to be more symptomatic and to perform worse in terms of pain and functional outcomes after surgical interventions [37,38,39,40]. The subchondral bone changes that accompany higher grade defects may include edema or cysts, which appear hyperintense on fluid-sensitive sequences, or fibrous/sclerotic changes, which appear hypointense. Associated changes in the joint may include synovitis, effusion and osteophytes.

Various other classification systems have been proposed, which are used in clinical trials to provide a semiquantitative scoring assessment. These include the Whole-Organ MRI Score (WORMS), the Knee Osteoarthritis Scoring System (KOSS), the Boston–Leeds Osteoarthritis Knee Score (BLOKS) and the MRI Osteoarthritis Knee Score (MOAKS) [41,42,43,44]. These systems divide the knee into various (nine to fourteen) sub-regions, and the cartilage defects are quantified using different scoring systems. Associated changes in the joint as well as meniscal and ligamentous abnormalities are also assessed. These are complex and time-consuming classifications and thus find very little utility in a busy clinical scenario. Colak et al. compared the MOAKS semiquantitative scoring system with the modified Outerbridge classification and found both to be effective for predicting outcomes after partial meniscectomy, with the latter showing better inter-reader agreement [45].

While there are a number of MRI sequences available, 3D T1-weighted SPGR (spoiled gradient echo) and FSE (fast spin echo) sequences are the most widely used for cartilage assessment [46,47]. Fat-suppressed 3D SPGR sequences are highly sensitive for the detection of cartilage defects. The disadvantages, however, include a relatively long acquisition time and sensitivity to artifacts, which may compromise image quality in post-operative patients. FSE sequences, in addition to having a high sensitivity for the detection of cartilage abnormalities, have the advantage of a shorter scan time as well as the ability to assess other joint structures such as ligaments and menisci [48,49]. 

**Figure 9:**
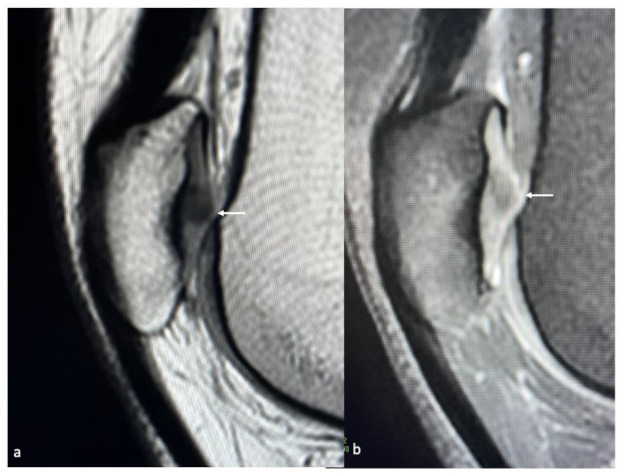
Sagittal PD (**a**) and PDFS images (**b**) showing chondropathy of the articular cartilage of the patella (arrow).

**Figure 10:**
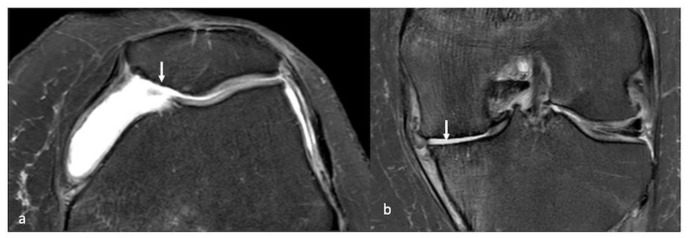
Axial PDFS images (**a**) showing a full-thickness cartilage defect of the patella (arrow) and (**b**) full-thickness cartilage defects of the medial tibiofemoral joint (arrow).

**Figure 11:**
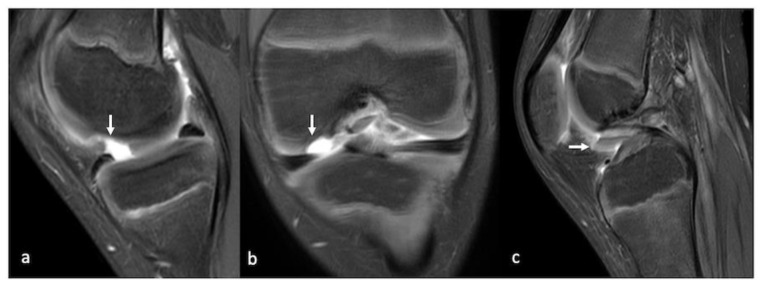
PDFS sagittal (**a**), coronal (**b**) and sagittal (**c**) images of the knee showing a full-thickness chondral defect of the medial femoral condyle (arrow) with a displaced chondral fragment in the anterior recess (arrow).

As discussed, the early or low-grade lesions may manifest as areas of increased signal intensity on fluid-sensitive sequences without surface defects representing cartilage softening, or as superficial blisters and fibrillation. Higher grade lesions appear as T2 hyperintense fluid-filled gaps which may have obtuse margins if they are chronic or acute margins if they are acute/traumatic in nature (Figure 9, Figure 10 and Figure 11).

**Figure 12:**
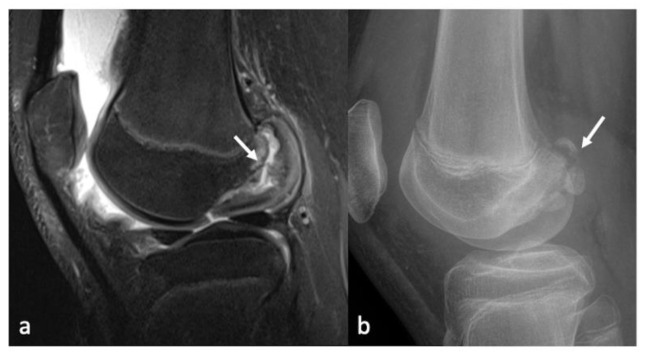
PDFS sagittal (**a**) and lateral radiographs of the knee (**b**) showing unstable OCD of the lateral femoral condyle (arrow).

Cartilage defects may occur in isolation or along with defects of the subchondral bone, in which case they are called osteochondral defects (Figure 12). Various etiologies that may give rise to osteochondral lesions apart from osteoarthritis include trauma, osteochondritis dissecans (OCD), collapse of the subchondral bone due to insufficiency fractures or avascular necrosis [50]. A detailed discussion about the imaging differences of these entities is beyond the scope of this article. However, an appropriate history examination along with MR imaging can help differentiate the etiology in most cases. Prognostic factors include size, location, number of lesions, presence or absence of overlying cartilage, presence of viable or non-viable subchondral bone (in cases of avascular necrosis). MRI can also help localize detached intra-articular loose bodies or predict unstable lesions. The classic criteria for unstable lesions include (a) a high-intensity rim signal between the lesion and the parent bone on fluid-sensitive sequences; (b) cysts beneath the lesion; (c) a high signal intensity extending through the cartilage over the bone lesion (d) a focal fluid-filled defect of the bone suggesting complete detachment [50,51]. Surgical treatment is usually indicated in the absence of symptoms resolution with a conservative management or for unstable OCD lesions [52,53,54].

The grading of osteochondral lesions has evolved over time, from Berndt and Harty describing radiographic grading in 1959 [55,56], modified by Anderson in 1989 [56] and by Loomer in 1993 [57], to now include MRI features, as in the grading system proposed by Hepple [58] [Table 2].

## 5. Imaging Evaluation of Cartilage Repair Procedures

While the current management of osteoarthritis aims primarily to alleviate pain and improve joint function through non-pharmacological measures and the use of drugs, surgery is offered as the last resort. However, there have been many advances in the treatment of focal cartilage defects. These procedures are largely divided into local stimulation and autologous transplantation techniques.

With local stimulation techniques, the subchondral bone beneath the cartilage defect is penetrated to cause bleeding, leading to fibrin clot formation. This fibrin clot contains pluripotent cells that remodel into repair tissue, which is either fibrocartilage or a combination of fibrocartilage and hyaline cartilage. However, the long-term joint function sustainability by the fibrocartilage is probably limited owing to its inferior biomechanical properties compared to those of hyaline cartilage. The commoner techniques used to produce defects in the subchondral bone include abrasion arthroplasty, microfracture and subchondral drilling. 

The common autologous transplantation procedures include autologous osteochondral transplantation (AOT) and autologous chondrocyte implantation (ACI). The former includes harvesting cylindrical osteochondral plugs of variable sizes from the relatively non-weight-bearing areas of the joint and transplanting them to fill chondral defects. ACI is a two-step procedure where a small sample of cartilage is harvested from non-weight-bearing areas, and cells from this specimen are isolated and cultured for several weeks. In the second step, an arthrotomy is performed, and the periosteum harvested from another bone is used to cover the defect; the cultured cells are then injected beneath it. These techniques have the potential to form hyaline cartilage and are, thus, of great interest to researchers [49,59,60,61,62].

**Figure 13:**
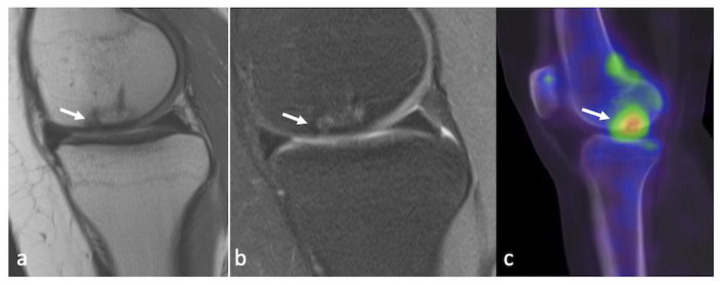
Sagittal PD (**a**), PDFS (**b**) and SEPCT images (**c**) showing post-OCD fixation changes with increased uptake (arrow).

Although the imaging results of these techniques vary with time, in about 1 to 2 years the defect should ideally be filled by the reparative tissue, or the autograft should be well taken up. On MRI, the signal intensity of the graft should match that of the native cartilage, with a failed graft appearing fragmented or irregular. Signs of the failure of the incorporation may be seen as the presence of a fluid signal between the graft and the native bone, subchondral cysts or sclerosis. A well-incorporated graft has indefectible margins, with smooth contours of the overlying cartilage (Figure 13).

The assessment of other areas of the joint is necessary, as the underlying disease process such as osteoarthritis may produce newer cartilage defects. The donor site for osteochondral defects should show a return to a normal fatty marrow signal intensity in less than a year, with the cartilage defect being filled with reparative tissue. Complications in the donor site may be seen in about 3% of the cases [63]. 

## 6. Conclusions

Articular cartilage pathologies are a significant cause of morbidity. Imaging, in particular MRI, has an established role in the diagnosis, quantification and prognostication of disease burden. With innovative advances in imaging, the biochemical and structural changes characterizing these pathologies are better appreciated and understood. 

## Figures and Tables

**Table 1:** Components of the cartilage.

Water	65–80% wet weight (80% in superficial and 65% in deep zone) Provides nutrition, reduces friction, allows deformation under loads.
Collagen	10–20% wet weight Type II collagen is the main component, provides tensile strength
Proteoglycans (PGs)	10–20% wet weight Provide compressive strength The subunits are called glycosaminoglycans (GAGs) Contain negatively charged groups that attract positively charged molecules (Na^+^). Maintain the fluid–electrolyte balance and increase the osmolarity of cartilage
Chondrocytes	Form only 1–5% of the volume Produce the components of the matrix and maintain metabolism Dependent on anaerobic metabolism

**Table 2:** Some of the popular grading systems for osteochondral lesions.

Berndt and Harty (X-ray)	Loomer et al. [57] Modification (CT)	Hepple et al. [58] (MRI)
(I) Trabecular compression of subchondral bone (II) Partially detached osteochondral fragment (III) Completely detached but undisplaced lesion (IV) Detached and displaced lesion	(I) Compressed (II) Chip avulsed but attached (III) Detached chip but undisplaced (IV) Detached and displaced chip (V) Radiolucent, cystic lesion	(1) Articular cartilage damage only (2a) Cartilage injury with underlying fracture and surrounding bony edema (2b) Stage 2a without surrounding bony edema (3) Detached but undisplaced fragment (4) Detached and displaced fragment (5) Subchondral cyst formation

## Data Availability

Not Applicable.

## References

[1] Cui A., Li H., Wang D., Zhong J., Chen Y., Lu H. (2020). Global, regional prevalence, incidence and risk factors of knee osteoarthritis in population-based studies. Eclinicalmedicine.

[2] Sophia Fox A.J., Bedi A., Rodeo S.A. (2009). The Basic Science of Articular Cartilage: Structure, Composition, and Function. Sport. Health A Multidiscip. Approach.

[3] Bhosale A.M., Richardson J.B. (2008). Articular cartilage: Structure, injuries and review of management. Br. Med. Bull..

[4] Alford J.W., Cole B.J. (2005). Cartilage Restoration, Part 1. Am. J. Sport. Med..

[5] Decker R.S., Koyama E., Pacifici M. (2015). Articular Cartilage: Structural and Developmental Intricacies and Questions. Curr. Osteoporos. Rep..

[6] Mollenhauer J., Aurich M.E., Zhong Z., Muehleman C., Cole A.A., Hasnah M., Oltulu O., Kuettner K.E., Margulis A., Chapman L.D. (2002). Diffraction-enhanced X-ray imaging of articular cartilage. Osteoarthr. Cartil..

[7] Aisen A.M., McCune W.J., Macguire A., Carson P.L., Silver T.M., Jafri S.Z., Martel W. (1984). Sonographic evaluation of the cartilage of the knee. Radiology.

[8] Lee C.-L., Huang M.-H., Chai C.-Y., Chen C.-H., Su J.-Y., Tien Y.-C. (2008). The validity of in vivo ultrasonographic grading of osteoarthritic femoral condylar cartilage: A comparison with in vitro ultrasonographic and histologic gradings. Osteoarthr. Cartil..

[9] Maeguchi K., Ito H., Morita Y., Furu M., Fujii T., Azukizawa M., Okahata A., Nishitani K., Kuriyama S., Nakamura S. (2018). How precisely does ultrasonographic evaluation reflect the histological status of the articular cartilage of the knee joint?. J Orthop..

[10] Iagnocco A. (2010). Imaging the joint in osteoarthritis: A place for ultrasound?. Best Pract. Res. Clin. Rheumatol..

[11] Meenagh G., Iagnocco A., Filippucci E., Riente L., Sedie A.D., Bombardieri S., Valesini G., Grassi W. (2006). Ultrasound imaging for the rheumatologist IV. Ultrasonography of the knee. Clin. Exp. Rheumatol..

[12] Vlad V., Micu M., Porta F., Radunovic G., Nestorova R., Petranova T., Cerinic M.M., Iagnocco A. (2012). Ultrasound of the hand and wrist in rheumatology. Med. Ultrason..

[13] Roemer F.W., Demehri S., Omoumi P., Link T.M., Kijowski R., Saarakkala S., Crema M.D., Guermazi A. (2020). State of the Art: Imaging of Osteoarthritis—Revisited 2020. Radiology.

[14] Yoo H.J., Hong S.H., Choi J.-Y., Lee I.J., Kim S.J., Kang H.S. (2011). Contrast-enhanced CT of Articular Cartilage: Experimental Study for Quantification of Glycosaminoglycan Content in Articular Cartilage. Radiology.

[15] Carrino J.A., al Muhit A., Zbijewski W., Thawait G.K., Stayman J.W., Packard N., Senn R., Yang D., Foos D.H., Yorkston J. (2014). Dedicated Cone-Beam CT System for Extremity Imaging. Radiology.

[16] Demehri S., Muhit A., Zbijewski W., Stayman J.W., Yorkston J., Packard N., Senn R., Yang D., Foos D., Thawait G.K. (2015). Assessment of image quality in soft tissue and bone visualization tasks for a dedicated extremity cone-beam CT system. Eur. Radiol..

[17] Barr C., Bauer J.S., Malfair D., Ma B., Henning T.D., Steinbach L., Link T.M. (2006). MR imaging of the ankle at 3 Tesla and 1.5 Tesla: Protocol optimization and application to cartilage, ligament and tendon pathology in cadaver specimens. Eur. Radiol..

[18] Oei E.H., Wick M.C., Müller-Lutz A., Schleich C., Miese F.R. (2018). Cartilage Imaging: Techniques and Developments. Semin. Musculoskelet. Radiol..

[19] Eagle S., Potter H.G., Koff M.F. (2016). Morphologic and quantitative magnetic resonance imaging of knee articular cartilage for the assessment of post-traumatic osteoarthritis. J. Orthop. Res..

[20] Mamisch T.C., Trattnig S., Quirbach S., Marlovits S., White L.M., Welsch G.H. (2010). Quantitative T2 Mapping of Knee Cartilage: Dif-ferentiation of Healthy Control Cartilage and Cartilage Repair Tissue in the Knee with Unloading—Initial Results. Radiology.

[21] Mars M., Tbini Z., Gharbi S., Bouaziz M.C., Ladeb F. (2018). T2 Versus T2* MRI Mapping in the Knee Articular Cartilage at 1.5 Tesla and 3 Tesla. Open Med. J..

[22] Fleming B., Oksendahl H., Mehan W., Portnoy R., Fadale P., Hulstyn M., Bowers M., Machan J., Tung G. (2010). Delayed Gadolinium-Enhanced MR Imaging of Cartilage (dGEMRIC) following ACL injury. Osteoarthr. Cartil..

[23] Schmaranzer F., Haefeli P.C., Hanke M.S., Liechti E.F., Werlen S.F., Siebenrock K.A., Tannast M. (2017). How Does the dGEMRIC Index Change After Surgical Treatment for FAI? A Prospective Controlled Study: Preliminary Results. Clin. Orthop. Relat. Res..

[24] Watanabe A., Wada Y., Obata T., Ueda T., Tamura M., Ikehira H., Moriya H. (2006). Delayed Gadolinium-enhanced MR to Determine Gly-cosaminoglycan Concentration in Reparative Cartilage after Autologous Chondrocyte Implantation: Preliminary Results. Radiology.

[25] Stahl R., Luke A., Li X., Carballido-Gamio J., Ma C.B., Majumdar S., Link T.M. (2009). T1rho, T2 and focal knee cartilage abnormalities in physically active and sedentary healthy subjects versus early OA patients—A 3. 0-Tesla MRI study. Eur. Radiol..

[26] Li Z., Wang H., Lu Y., Jiang M., Chen Z., Xi X., Ding X., Yan F. (2019). Diagnostic value of T1ρ and T2 mapping sequences of 3D fat-suppressed spoiled gradient (FS SPGR-3D) 3.0-T magnetic resonance imaging for osteoarthritis. Medicine.

[27] Le J., Peng Q., Sperling K. (2016). Biochemical magnetic resonance imaging of knee articular cartilage: T1rho and T2 mapping as car-tilage degeneration biomarkers. Ann. N. Y. Acad. Sci..

[28] Bolbos R., Link T., Ma C.B., Majumdar S., Li X. (2009). T1ρ relaxation time of the meniscus and its relationship with T1ρ of adjacent cartilage in knees with acute ACL injuries at 3T. Osteoarthr. Cartil..

[29] Rakhra K.S., Melkus G., Anwander H., E Beaulé P. (2016). T1ρ MRI detects cartilage damage in asymptomatic individuals with a cam deformity. J. Orthop. Res..

[30] Wheaton A.J., Borthakur A., Shapiro E.M., Regatte R.R., Akella S.V.S., Kneeland J.B., Reddy R. (2004). Proteoglycan Loss in Human Knee Cartilage: Quantitation with Sodium MR Imaging—Feasibility Study. Radiology.

[31] Madelin G., Babb J.S., Xia D., Chang G., Jerschow A., Regatte R.R. (2012). Reproducibility and repeatability of quantitative sodium mag-netic resonance imaging in vivo in articular cartilage at 3 T and 7 T. Magn. Reson. Med..

[32] Kogan F., Hargreaves B.A., Gold G.E. (2016). Volumetric multislice gagCEST imaging of articular cartilage: Optimization and comparison with T1rho. Magn. Reson. Med..

[33] Schmitt B., Zbýň Š., Stelzeneder D., Jellus V., Paul D., Lauer L., Bachert P., Trattnig S. (2011). Cartilage Quality Assessment by Using Glycosaminoglycan Chemical Exchange Saturation Transfer and ^23^Na MR Imaging at 7 T. Radiology.

[34] Nakamura H., Masuko K., Yudoh K., Kato T., Nishioka K., Sugihara T., Beppu M. (2007). Positron emission tomography with 18F-FDG in osteoarthritic knee. Osteoarthr. Cartil..

[35] Kobayashi N., Inaba Y., Tateishi U., Yukizawa Y., Ike H., Inoue T., Saito T. (2013). New Application of 18F-Fluoride PET for the Detection of Bone Remodeling in Early-Stage Osteoarthritis of the Hip. Clin. Nucl. Med..

[36] Jena A., Taneja S., Rana P., Goyal N., Vaish A., Botchu R., Vaishya R. (2021). Emerging role of integrated PET-MRI in osteoarthritis. Skelet. Radiol..

[37] Sofu H., Oner A., Camurcu Y., Gursu S., Ucpunar H., Sahin V. (2016). Predictors of the Clinical Outcome After Arthroscopic Partial Meniscectomy for Acute Trauma–Related Symptomatic Medial Meniscal Tear in Patients More Than 60 Years of Age. Arthrosc. J. Arthrosc. Relat. Surg..

[38] Kemp J.L., Makdissi M., Schache A.G., Pritchard M.G., Pollard T.C.B., Crossley K.M. (2014). Hip chondropathy at arthroscopy: Prevalence and relationship to labral pathology, femoroacetabular impingement and patient-reported outcomes. Br. J. Sport. Med..

[39] Bateman D.K., Black E.M., Lazarus M.D., Abboud J.A. (2017). Outcomes Following Arthroscopic Repair of Posterior Labral Tears in Patients Older Than 35 Years. Orthopedics.

[40] Slattery C., Kweon C.Y. (2018). Classifications in Brief: Outerbridge Classification of Chondral Lesions. Clin. Orthop. Relat. Res..

[41] Peterfy C., Guermazi A., Zaim S., Tirman P., Miaux Y., White D., Kothari M., Lu Y., Fye K., Zhao S. (2004). Whole-Organ Magnetic Resonance Imaging Score (WORMS) of the knee in osteoarthritis. Osteoarthr. Cartil..

[42] Kornaat P.R., Ceulemans R.Y.T., Kroon H.M., Riyazi N., Kloppenburg M., Carter W.O., Woodworth T.G., Bloem J.L. (2004). MRI assessment of knee osteoarthritis: Knee Osteoarthritis Scoring System (KOSS)?inter-observer and intra-observer reproducibility of a compartment-based scoring system. Skelet. Radiol..

[43] Hunter D.J., Lo G.H., Gale D., Grainger A.J., Guermazi A., Conaghan P.G. (2008). The reliability of a new scoring system for knee osteo-arthritis MRI and the validity of bone marrow lesion assessment: BLOKS (Boston–Leeds Osteoarthritis Knee Score). Ann. Rheum. Dis..

[44] Hunter D., Guermazi A., Lo G., Grainger A., Conaghan P., Boudreau R., Roemer F. (2011). Evolution of semi-quantitative whole joint assessment of knee OA: MOAKS (MRI Osteoarthritis Knee Score). Osteoarthr. Cartil..

[45] Colak C., Polster J.M., Obuchowski N.A., Jones M.H., Strnad G., Gyftopoulos S., Spindler K.P., Subhas N. (2020). Comparison of Clinical and Semiquantitative Cartilage Grading Systems in Predicting Outcomes After Arthroscopic Partial Meniscectomy. Am. J. Roentgenol..

[46] Disler D.G., McCauley T.R., Wirth C.R., Fuchs M.D. (1995). Detection of knee hyaline cartilage defects using fat-suppressed three-dimensional spoiled gradient-echo MR imaging: Comparison with standard MR imaging and correlation with arthros-copy. Am. J. Roentgenol..

[47] Recht M.P., Piraino D.W., Paletta G.A., Schils J.P., Belhobek G.H. (1996). Accuracy of fat-suppressed three-dimensional spoiled gradi-ent-echo FLASH MR imaging in the detection of patellofemoral articular cartilage abnormalities. Radiology.

[48] Bredella M.A., Tirman P.F., Peterfy C.G., Zarlingo M., Feller J.F., Bost F.W., Belzer J.P., Wischer T.K., Genant H.K. (1999). Accuracy of T2-weighted fast spin-echo MR imaging with fat saturation in detecting cartilage defects in the knee: Comparison with arthroscopy in 130 patients. Am. J. Roentgenol..

[49] Recht M.P., Goodwin D.W., Winalski C.S., White L.M. (2005). MRI of Articular Cartilage: Revisiting Current Status and Future Directions. Am. J. Roentgenol..

[50] Gorbachova T., Melenevsky Y., Cohen M., Cerniglia B.W. (2018). Osteochondral Lesions of the Knee: Differentiating the Most Common Entities at MRI. Radiographics.

[51] Smet A.A.D., Ilahi O.A., Graf B.K. (1996). Reassessment of the MR criteria for stability of osteochondritis dissecans in the knee and ankle. Skeletal. Radiol..

[52] Kijowski R., de Smet A.A. (2005). MRI Findings of Osteochondritis Dissecans of the Capitellum with Surgical Correlation. Am. J. Roentgenol..

[53] Takahara M., Ogino T., Takagi M., Tsuchida H., Orui H., Nambu T. (2000). Natural Progression of Osteochondritis Dissecans of the Humeral Capitellum: Initial Observations. Radiology.

[54] Schenck R.C., Goodnight J.M. (1996). Osteochondritis dissecans. J. Bone Jt. Surg. Am..

[55] Berndt Al Harty M. (1959). Transchondral fractures (osteochondritis dissecans) of the talus. J. Bone Jt. Surg. Am..

[56] Anderson I.F., Crichton K.J., Grattan-Smith T., Cooper R.A., Brazier D. (1989). Osteochondral fractures of the dome of the talus. J. Bone Jt. Surg. Am..

[57] Loomer R., Fisher C., Lloyd-Smith R., Sisler J., Cooney T. (1993). Osteochondral lesions of the talus. Am. J. Sport. Med..

[58] Hepple S., Winson I.G., Glew D. (1999). Osteochondral Lesions of the Talus: A Revised Classification. Foot Ankle Int..

[59] Choi Y.S., Potter H.G., Chun T.J. (2008). MR Imaging of Cartilage Repair in the Knee and Ankle. Radiographics.

[60] Verstraete K., Almqvist F., Verdonk P., Vanderschueren G., Huysse W., Verdonk R., Verbrugge G. (2004). Magnetic resonance imaging of cartilage and cartilage repair. Clin. Radiol..

[61] Alparslan L., Winalski C.S., Boutin R.D., Minas T. (2001). Postoperative Magnetic Resonance Imaging of Articular Cartilage Repair. Semin. Musculoskelet. Radiol..

[62] Smith G.D., Knutsen G., Richardson J.B. (2005). A clinical review of cartilage repair techniques. J. Bone Jt. Surg. Am..

[63] Hangody L., Ráthonyi G.K., Duska Z., Vásárhelyi G., Füles P., Módis L. (2004). Autologous osteochondral mosaicplasty: Surgical technique. J. Bone Jt. Surg. Am..

